# Frequent Transmission of Streptococcus pneumoniae Serotype 35B and 35D, Clonal Complex 558 Lineage, across Continents and the Formation of Multiple Clades in Japan

**DOI:** 10.1128/aac.01083-22

**Published:** 2023-01-18

**Authors:** Koh Shinohara, Takao Fujisawa, Bin Chang, Yutaka Ito, Shigeru Suga, Yasufumi Matsumura, Masaki Yamamoto, Miki Nagao, Makoto Ohnishi, Motoyuki Sugai, Satoshi Nakano

**Affiliations:** a Department of Clinical Laboratory Medicine, Kyoto University Graduate School of Medicine, Kyoto, Japan; b National Hospital Organization Mie National Hospital, Tsu, Japan; c Bacteriology I, National Institute of Infectious Diseases, Tokyo, Japan; d Nagoya City University, Graduate School of Medical Science, Nagoya, Japan; e Antimicrobial Resistance Research Center, National Institute of Infectious Diseases, Tokyo, Japan

**Keywords:** *Streptococcus pneumoniae*, pneumococcal disease, serotype 35B, serotype 35D, multidrug resistance, whole-genome sequencing

## Abstract

Streptococcus pneumoniae is a common bacterial pathogen that causes infections in children worldwide, even after administration of the pneumococcal conjugate vaccine. S. pneumoniae serotype 35B, especially the clonal complex 558 (CC558) lineage, has emerged globally following implementation of the 13-valent pneumococcal conjugate vaccine. Serotype 35B strains are also associated with multidrug resistance to both β-lactams and non-β-lactam drugs. In addition, a novel serotype, 35D, which is closely related to 35B and differs in polysaccharide structure, was recently reported. However, the genetic relationship among globally disseminating serotype 35B and D (35B/D) strains remains unknown. To investigate the molecular epidemiology of global serotype 35B/D strains, we conducted a genomic analysis of serotype 35B/D strains from various continents, including those from the Japanese national surveillance collection. A total of 87 isolates were identified as serotype 35B/D in the Japanese surveillance collection (*n* = 1,358). All the isolates were assigned to either CC558 or CC2755. Serotype 35D isolates were interspersed with serotype 35B isolates. Phylogenetic analysis revealed the formation of multiple clusters by the Japanese serotype 35B/D-CC558 isolates among the foreign isolates, which suggested multiple events of introduction of the clone into Japan. The global 35B/D-CC558 strains were found to share specific penicillin-binding protein profiles, *pbp1a*-4, *pbp2b*-7, and *pbp2x*-7, associated with penicillin, cephalosporin, and carbapenem nonsusceptibility. Moreover, 88.5% of the Japanese 35B/D-CC558 and 35B/D-CC2755 isolates were found to harbor the Tn*916*-like integrative and conjugative elements Tn*2009*, Tn*2010*, and Tn*6002*, associated with multidrug resistance to macrolides and tetracyclines. The results of this study imply that serotype 35B/D-CC558 strains could be frequently transmitted intercontinentally.

## INTRODUCTION

Streptococcus pneumoniae is a common bacterial pathogen that causes infections in children worldwide despite the introduction of pneumococcal conjugate vaccines (PCVs) in many countries (1). The introduction of PCVs resulted in a universal decline in the incidence of vaccine serotype invasive pneumococcal diseases (IPDs) by over 90% (2, 3). However, replacement by non-PCV serotypes has negated the overall effect of vaccination on IPDs in several regions (4, 5). In the post-13-valent PCV (PCV13) era, non-PCV serotypes 12F, 15B and 15C (15B/C), and 35B/D are among the most prevalent, with geographical expansion across the United States, Eurasia, and Africa in a global surveillance study using whole-genome sequencing (6).

IPDs caused by serotype 35B were rare before the introduction of PCVs. In the United States, serotype 35B accounted for only 0.5% of IPDs before the introduction of PCV7 (2). However, in the PCV era, the prevalence of serotype 35B in both IPD and nasopharyngeal carriage has been increasing in the United States (3, 7), and this trend has also been observed in other countries (8). The increasing prevalence of serotype 35B is of great concern because it is associated with high rates of penicillin resistance (9). Serotype 35B is also associated with a high risk of death due to IPD (10). In particular, the 35B-clonal complex 558 (CC558) lineage was reported as one of the major contributors to the increase in the incidence of multidrug-resistant IPDs after PCV7 and PCV13 implementation in the United States (11).

This lineage also served as a donor during capsular switching of sequence type 156 (ST156) strains, resulting in the emergence of nonvaccine type and multidrug-resistant (MDR) serotype 35B-ST156 strains (12). Thus, the emergence and spread of antibiotic-resistant S. pneumoniae strains are of great concern because they are associated with poor clinical outcomes and increased health care costs; therefore, continuous monitoring of such lineages is important (13).

PCV7 and PCV13 were introduced in Japan in 2010 and 2013, respectively. An increase in the incidence of IPDs caused by non-PCV serotypes, including 35B, has also been observed after PCV introduction in Japan (14, 15). Kasahara et al. reported a 3% (19/641) prevalence of 35B among pneumococcal isolates obtained between 2002 and 2012 in Japan (16). Among these, 12/19 (63.2%) were ST558 and 7/19 (36.8%) were ST2755. After the introduction of PCV13, serotype 35B reportedly accounted for 11.0% of all pneumococcal isolates from IPD and non-IPD patients (15). CC558 isolates, all of which were penicillin resistant, accounted for 77.9% of serotype 35B isolates, whereas ST2755 accounted for 19.8% of the serotype (15). Moreover, an increase in the frequency of MDR strains resistant to β-lactams, including penicillin, and non-β-lactams, such as macrolides, tetracyclines, and trimethoprim-sulfamethoxazole, has also been reported (16–18). However, the genetic relationship between global 35B-CC558 isolates and factors attributed to the dissemination of MDR serotype 35B strains is unclear.

Recently, a newly recognized serotype, 35D, which differs from 35B polysaccharide in structure and serology, was reported (19). Serotype 35D strains lack *O*-acetylation of capsular polysaccharides, an antigenically dominant epitope, due to a deficient *O*-acetyltransferase encoded by *wgiG*; therefore, there is concern that these strains might escape the effect of future vaccines targeting serotype 35B (19). Furthermore, serotype 35D isolates have been reported globally, and CC558, CC156, and CC198 were the predominant CCs found in this serotype (20). Therefore, serotype 35D is speculated to have emerged sporadically from the closely related serotype 35B strains (20).

We investigated the molecular epidemiology of global serotype 35B and 35D strains, including those from a Japanese nationwide surveillance collection.

## RESULTS

### Whole-genome-sequencing statistics.

The whole-genome-sequencing statistics are shown in Data Set S3 in the supplemental material. The average (±standard deviation) number of contigs was 92.0 (±38.4), the *N*_50_ (shortest contig length needed to cover 50% of the genome) was 84,113 (±9,366), and the mapping depth was 121.7 (±52.5).

### STs, serotypes, and antimicrobial susceptibility.

**FIG 1:**
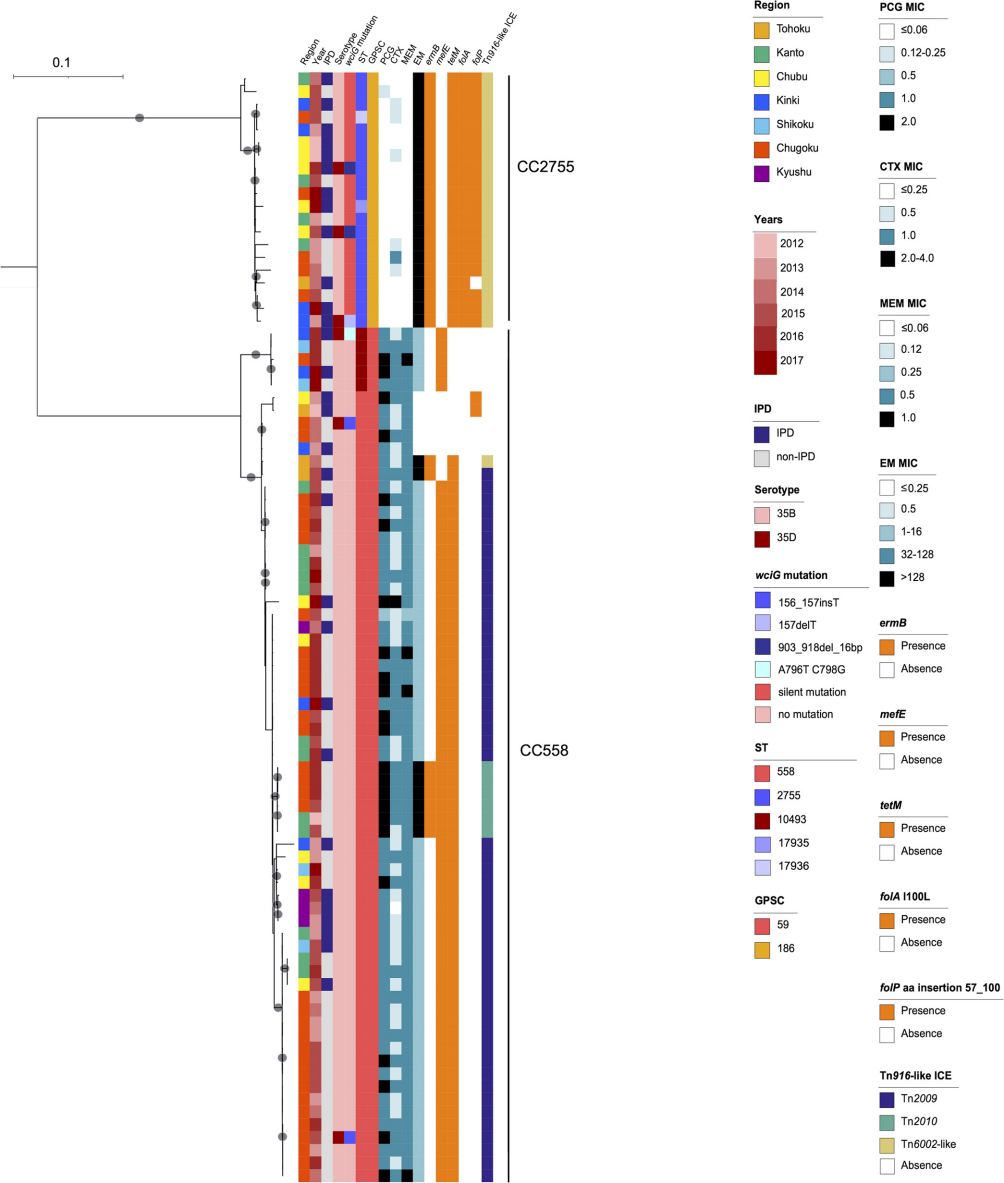
Phylogenetic tree of the serotype 35B and 35D isolates in Japan, generated by using RAxML-NG. Colored bars indicate characteristics of isolates including the region of isolation, year of isolation, serotype, susceptibility to penicillin, cefotaxime, meropenem, and erythromycin, presence of antimicrobial resistance determinants *ermB*, *tetM*, *folA* I100L mutation, and *folP* insertion, and types of Tn*916*-like integrative elements. Gray circles indicate ≥ 95% confidence by bootstrap analysis.

Among the 87 isolates originally typed as serotype 35B, we identified five sequence types (STs): ST558 (*n* = 62); ST10493 (*n* = 5), which is a single-locus variant (SLV) of ST558; ST2755 (*n* = 18); and ST17935 (*n* = 1) and ST17936 (*n* = 1), which are SLVs of ST2755. ST558 and ST10493 were assigned to CC558 and global pneumococcal sequence cluster 59 (GPSC59), whereas ST2755, ST17935, and ST17936 were assigned to CC2755 and GPSC186 (Fig. 1). Phylogenetic analysis of the Japanese serotype 35B and 35D isolates suggested a distant genetic relationship between CC558 and CC2755 (Fig. 1). Serotype 35D isolates were interspersed with serotype 35B isolates, irrespective of their CCs (Fig. 1).

The *wciG* alignment revealed that 63 isolates (72.4%) were identical to the serotype 35B reference (accession number KX021817), 21 isolates had silent mutations (G657A [a change of G to A at position 657] or C795T), and 6 isolates (6.9%) had either frameshift or nonsense mutations and were genotypically typed as serotype 35D (Table 1). Quellung reactions confirmed the phenotype of all 81 serotype 35B isolates. Among the six isolates genotypically typed as serotype 35D, three were phenotypically typed as serotype 35D, two as serotype 35B, and one as a mixture of both serotypes (Table 1). Moreover, among the 81 serotype 35B isolates, 26 (29.9%) were collected from IPD cases, compared with 4/6 (66.7%) of the serotype 35D isolates. Furthermore, three (50%) of the six serotype 35D isolates belonged to CC558 and the other three to CC2755 (Fig. 1). Significantly more CC2755 isolates originated from IPD cases than did CC558 isolates (12/20 [60.0%] versus 18/67 [26.9%], respectively; ~ odds ratio [OR], 4.08; 95% confidence interval [CI], 1.46 to 11.38; *P* = 0.014). Antimicrobial susceptibility results are summarized in Table 2. Overall, the rates of nonsusceptibility to penicillin G (PCG), cefotaxime (CTX), meropenem (MEM), erythromycin (EM), and levofloxacin (LFX) were 78.2%, 40.2%, 75.9%, 94.3%, and 0%, respectively. CC558 showed significantly higher rates of nonsusceptibility to PCG, CTX, and MEM. Of 82 EM-resistant isolates, 28 (34.1%) showed extremely high MICs (>128 mg/L) for EM (Fig. 1).

**TABLE 1:** Profiles of six genotypically serotype 35D S. pneumoniae isolates identified in this study

Strain	Prefecture (region)	Yr isolated	IPD/non-IPD^*a*^	Origin	Sequence type (ST)	*wciG* mutation(s)	Type of mutation	Reaction to antiserum^*b*^								Phenotypic serotype^*c*^
								Pool G	Type 29	Group 35	fs35a	fs35b	fs35c	fs29b	fs42a	
PC0204	Osaka (Kinki)	2013	IPD	Blood	2755	157delT	Frameshift	+	+	+	+	−	+	+	−	35B/D
PC0607	Yamaguchi (Chugoku)	2014	Non-IPD	Middle-ear fluid	558	156_157insT	Frameshift	+	+	+	+	−	+	+	−	35B
PC0780	Yamaguchi (Chugoku)	2015	Non-IPD	Middle-ear fluid	558	156_157insT	Frameshift	+	+	+	+	−	+	+	−	35B
PC0870	Nagano (Chubu)	2015	IPD	Blood	2755	903_918del_16bp	Frameshift	+	+	+	−	−	+/−^*d*^	+	−	35D
PC1044	Aichi (Chubu)	2016	IPD	CSF^*e*^	2755	903_918del_16bp	Frameshift	+	+	−	−	−	+	+	−	35D
PC1147	Hyogo (Kinki)	2016	IPD	Blood	10493	A796T, C798G	Nonsense	+	+	−	−	−	+	+	−	35D

a IPD, invasive pneumococcal disease.

b Serotyping was performed using pneumococcal typing antisera (Statens Serum Institut, Copenhagen, Denmark).

c Phenotypic serotypes were determined based on the criteria described by Lo et al. (20).

d +/−, under the microscope, cells derived from a single-colony overnight culture showed both positive and negative effects on the antisera tested.

e CSF, cerebrospinal fluid.

**TABLE 2:** Antimicrobial susceptibility according to clonal complex

Antimicrobial^*a*^	No. of nonsusceptible isolates (%) in^*b*^:						Total (*n* = 87)	*P* value for CC558 vs CC2755^*c*^
	CC558		Total CC558 (*n* = 67)	CC2755		Total CC2755 (*n* = 20)		
	ST558 (*n* = 62)	ST10493 (*n* = 5)		ST2755 (*n* = 18)	Others (*n* = 2)			
PCG	62 (100)	5 (100)	67 (100)	1 (5.6)	0 (0)	1 (0.5)	68 (78.2)	<0.001
CTX	30 (48.4)	4 (80.0)	34 (50.7)	1 (5.6)	0 (0)	1 (0.5)	35 (40.2)	<0.001
MEM	61 (98.4)	5 (100)	66 (98.5)	0 (0)	0 (0)	0 (0)	66 (75.9)	<0.001
EM	57 (91.9)	5 (100)	62 (92.5)	18 (100)	2 (100)	20 (100)	82 (94.3)	0.585
LFX	0 (0)	0 (0)	0 (0)	0 (0)	0 (0)	0 (0)	0 (0)	1.000

a PCG, penicillin G; CTX, cefotaxime; MEM, meropenem; EM, erythromycin; LFX, levofloxacin.

b CC, clonal complex; ST, sequence type.

c A *P* value of <0.05 was considered statistically significant.

### PBP type and antimicrobial resistance genes.

**FIG 2:**
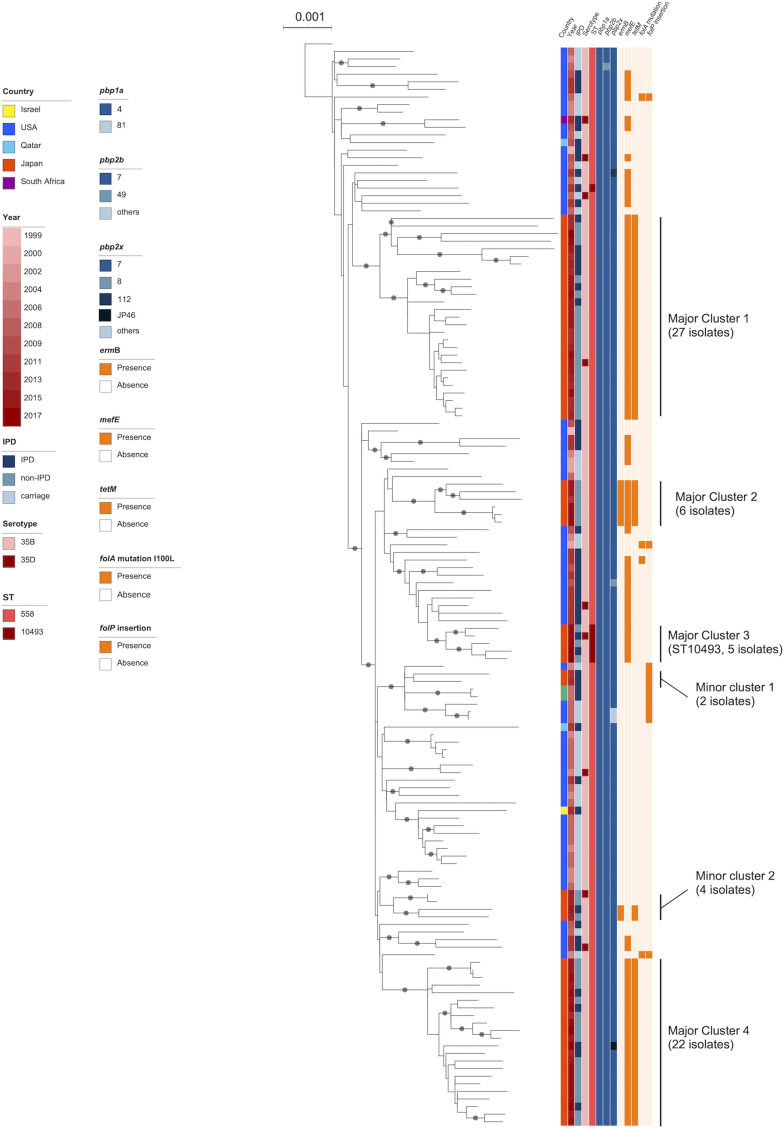
Recombination-free maximum-likelihood tree of the GPSC59 isolates in Japan and the previously deposited isolates in the NCBI database, rooted by the serotype 35B reference strain Utah35B (ST377, BAA-660TM [ATCC], accession no. AP025939). Colored bars indicate characteristics of isolates including the country of isolation, year of isolation, host status, serotype, sequence type, type of *pbp1a*, *php2b*, and *pbp2x*, and presence of antimicrobial resistance determinants *ermB*, *tetM*, *folA* I100L mutation, and *folP* insertion. Gray circles indicate ≥ 95% confidence by bootstrap analysis.

All CC558 (*n* = 67) isolates had penicillin-binding protein (PBP) genes *pbp1a*-4 and *pbp2b*-7, and 66 of 67 (98.5%) isolates had *pbp2x*-7, whereas one had *pbp2x*-JP46 (Fig. 2). All CC2755 (*n* = 20) isolates had *pbp1a*-0, and 19 of these had *pbp2b-*0, whereas one isolate had a novel type of *pbp2b*. Various *pbp2x* types were observed; JP34 (*n* = 5), JP36 (*n* = 3), and JP37 (*n* = 3) were the predominant types of *pbp2x* (Data Set S1).

The prevalence of antimicrobial resistance determinants in serotypes 35B and 35D, according to CC, is shown in Table 3. The number of isolates with macrolide resistance mediated by *ermB*, which showed extremely high MICs for EM, was significantly higher in CC2755 than in CC558 (CC558, 8/67 [11.9%]; CC2755, 20/20 [100%]; *P < *0.001). In contrast, macrolide resistance mediated by *mefE* was significantly higher in CC558 than in CC2755 (CC558, 60/67 [89.6%]; CC2755, 0/20 [0%]; *P < *0.001). The *folA* substitution and *folP* insertion mutations, to which full trimethoprim-sulfamethoxazole resistance is attributed, were exclusively found in CC2755. All CC558 isolates were *rrgC* (type 1 pilus [pilus-1]) positive and *sipA* (pilus-2) negative, and all CC2755 isolates were negative for both genes.

**TABLE 3:** Prevalence of antimicrobial resistance determinant genes according to serotype and clonal complex

Antimicrobial resistance determinant(s)	No. of isolates (%) according to serotype and clonal complex						*P* value for^*a*^:	
	35B (*n* = 81)		Total 35B	35D (*n* = 6)		Total 35D	35B vs 35D	CC558 vs CC2755
	CC558 (*n* = 64)	CC2755 (*n* = 17)		CC558 (*n* = 3)	CC2755 (*n* = 3)			
*ermB*	8 (12.5)	17 (100)	25 (30.9)	0 (0)	3 (100)	3 (50.0)	0.382	<0.001
*mefE*	58 (90.6)	0 (0)	58 (71.6)	2 (66.7)	0 (0)	2 (33.3)	0.072	<0.001
*tetM*	56 (87.5)	17 (100)	73 (90.1)	2 (66.7)	3 (100)	5 (83.3)	0.307	0.111
*folA* I100L and *folP* insertion	0 (0)	16 (94.1)	16 (19.8)	0 (0)	3 (100)	3 (50.0)	0.115	<0.001

a A *P* value of <0.05 was considered statistically significant.

### Phylogenetic analysis of serotype 35B and 35D isolates in Japan and other countries.

The phylogenetic tree of the global ST558 and ST10493 strains in GPSC59 revealed that the Japanese isolates formed four major clusters and two minor clusters interspersed among the global strains, mainly those from the United States (Fig. 2). ST10493 isolates from Japan, which formed major cluster 3 (Fig. 2), appeared to diverge from ST558 strains from the United States and not the Japanese strains. The phylogenetic tree of GPSC186 showed that the Japanese clade was genetically distinct from the previously deposited strains from other countries, whereas all GPSC186 strains were from Asian countries, and uniformly possessed *ermB*, *tetM*, and *folA* substitution and *folP* insertion mutations (Fig. S1).

### Tn*916*-like ICE structure and *cps* locus analysis.

**FIG 3:**
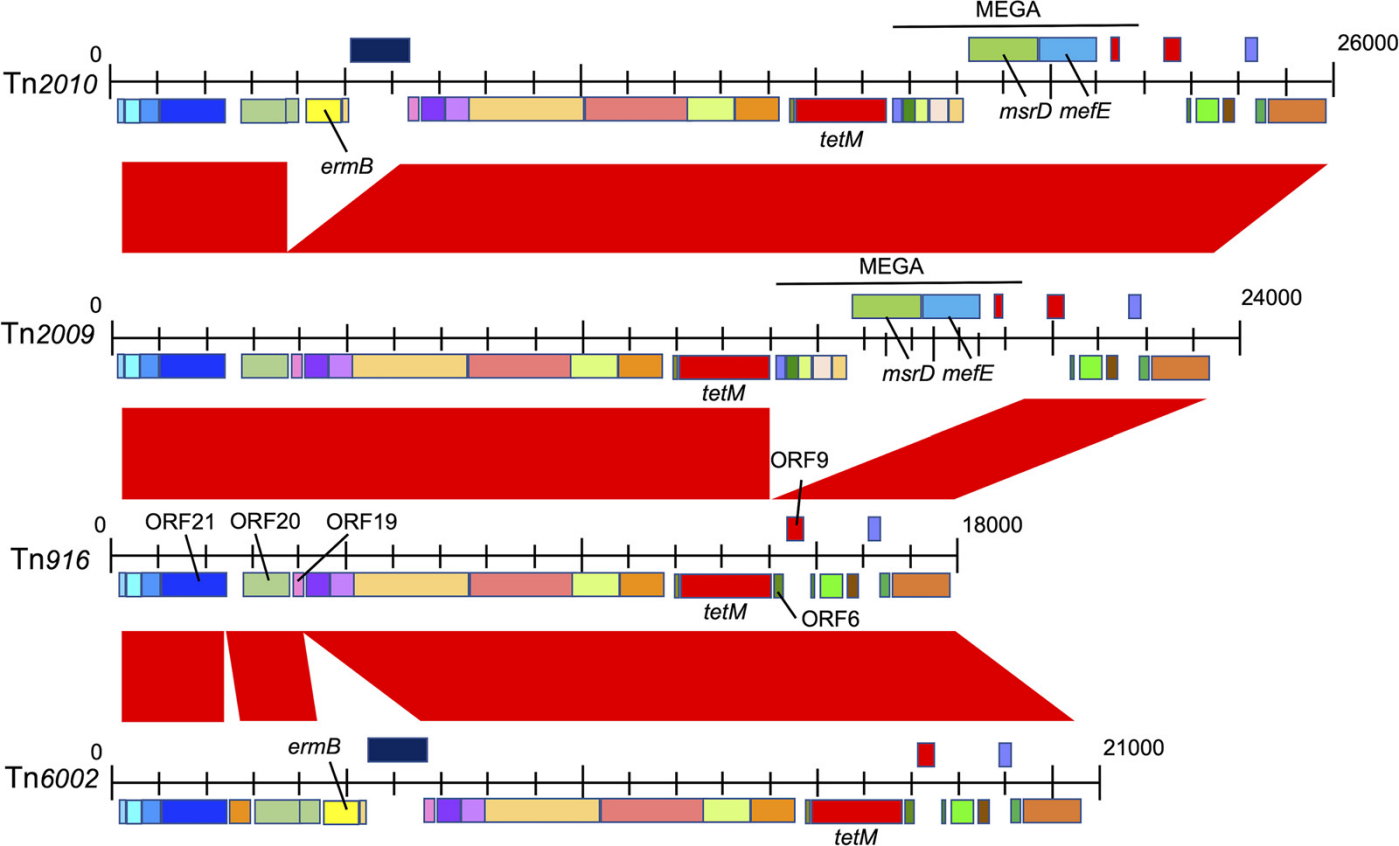
Comparisons of the Tn*916*-like integrative conjugative elements (ICEs) of serotype 35B/35D isolates. Red bands between the sequences indicate BLASTN matches. In Tn*2009*, a macrolide efflux genetic assembly (MEGA) that contained *mefE* was inserted between ORF6 and ORF9 of Tn*916*. Tn*2010*, in addition to the insertion of the MEGA in the same spot as Tn*2009*, had an *ermB*-containing cassette that was integrated with ORF20 of Tn*916*. Tn*6002* had an *ermB*-containing cassette that was integrated with ORF20 of Tn*916*, and *mefE* was not identified. The reference sequence of Tn*916* was submitted as NCBI reference sequence U09422.1.

Of the total study isolates, 77 (88.5%) had Tn*916*-like integrative and conjugative elements (ICEs) with *tetM*, which encodes tetracycline resistance (Fig. 3). Of the 67 CC558 isolates, 50 (74.6%) had Tn*2009*, which contains a macrolide efflux genetic assembly (MEGA) element that carries *mefE*. The MEGA element is inserted between open reading frame 6 (ORF6) and ORF9 of Tn*916*. Six (9.0%) of the CC558 isolates had Tn*2010*, which contains erythromycin resistance cassettes, including *ermB*, located between ORF20 and ORF21 of Tn*2009*. All ST2755 isolates and one CC558 isolate had Tn*6002*-like ICEs that contained erythromycin resistance cassettes, including *ermB*, between ORF19 and ORF20 and a hypothetical protein between ORF20 and ORF21 of Tn*916*, whereas *mefE* was not identified. None of the five ST10493 (CC558) isolates had Tn*916*-like ICEs, but they had the MEGA alone.

We compared the *cps* loci and *wciG* sequences of the ST558 isolate (PC0780; genotypically 35D and phenotypically 35B), the ST2755 isolate (PC0204; genotypically 35D and phenotypically 35B/D), and the reference genome of the *cps* locus of serotype 35B (accession number CR931705.1) (Fig. S2). The *cps* locus of PC0780 was consistent with that of the reference genome (accession number CR931705.1), whereas that of PC0204 contained three additional insertion sequences (ISs). We also evaluated the locations of the mutations/insertions/deletions in *wciG* of our isolates and previously reported serotype 35D isolates (20). The locations of the mutations/insertions/deletions were associated with the low-GC-content (AT-rich) regions in *wciG* (Fig. S3).

### Divergence time estimation.

**FIG 4:**
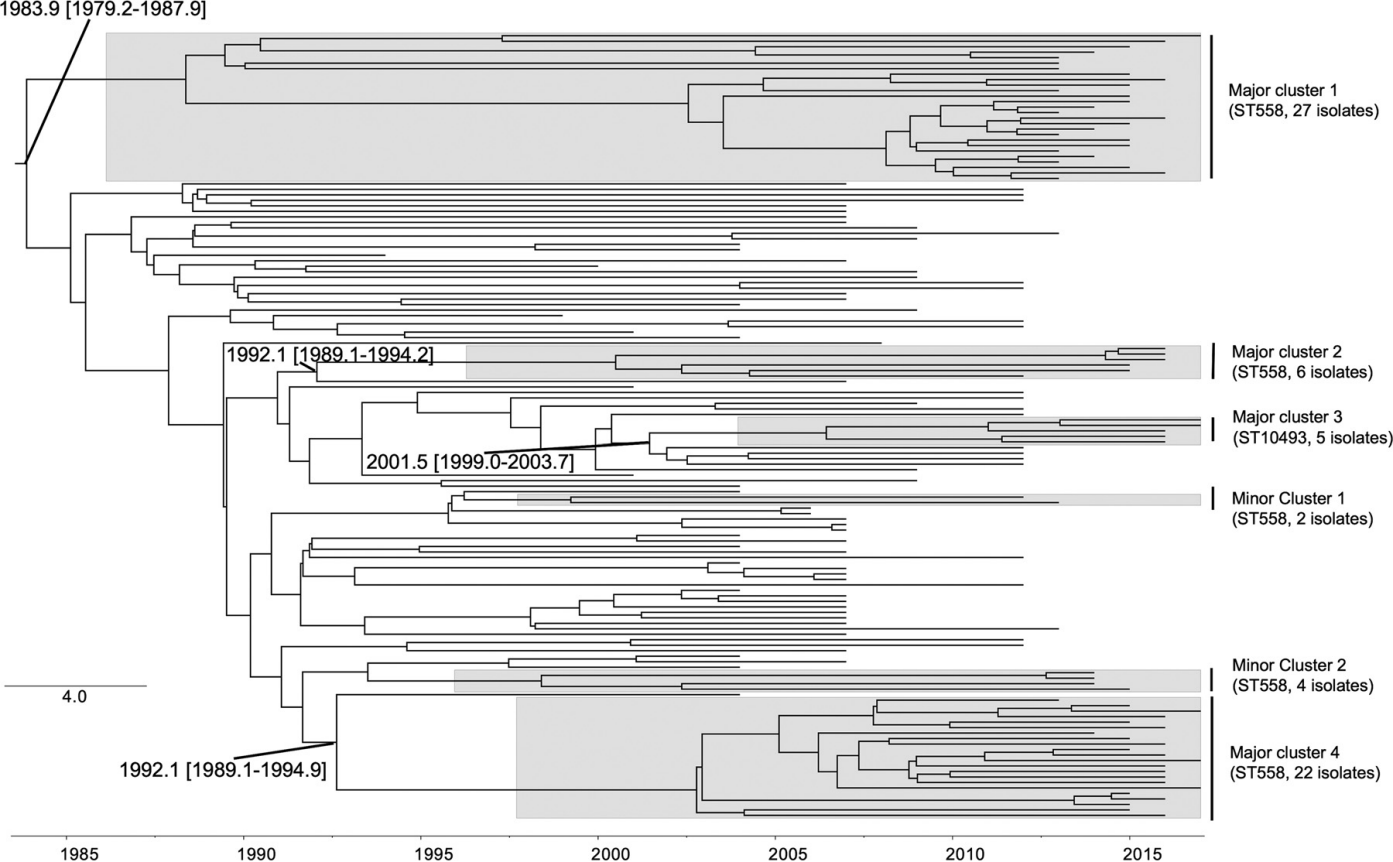
Bayesian phylogenetic reconstruction of ST558 and ST10493 isolates in GPSC59 from Japan and other countries, based on isolates. Major clusters (≥ 5 isolates) and minor clusters (< 5 isolates) containing only Japanese isolates are highlighted in gray. The estimated times of the most recent common ancestor of each major cluster and its 95% HPD are displayed.

We next performed Bayesian analysis to estimate the time to the most recent common ancestor (TMRCA) of the ST558 and ST10493 strains with serotype 35B/D, using BEAST2 (21). The TMRCAs of the four major clusters (containing ≥ 5 isolates) of these strains in Japan (major clusters 1 to 4) (Fig. 2 and 4), estimated using corresponding global GPSC59 strains, were 1983 (major cluster 1; 95% highest posterior density [HPD], 1979 to 1987), 1992 (major cluster 2; 95% HPD, 1989 to 2006), 2001 (major cluster 3; 95% HPD, 1999 to 2003), and 1992 (major cluster 4; 95% HPD, 1989 to 1994) (Fig. 4).

## DISCUSSION

Serotype 35B has become one of the most prevalent pneumococcal serotypes globally after the introduction of PCV13 (3, 6, 8). In addition, CC558 strains detected in various regions worldwide have exhibited multidrug resistance (12, 16, 17). This motivated us to analyze the genetic data of serotype 35B strains to understand their global spread after the introduction of PCV7 and -13, using whole-genome sequencing of serotype 35B strains isolated from four countries, the United States, Israel, Qatar, and South Africa (22–26), and compare them with those of strains isolated in Japan through a 6-year nationwide pneumococcal surveillance study. In addition, we assessed the phenotypic and genetic basis of the newly recognized serotype 35D, a serotype 35B variant, which is reportedly distributed globally, and suggested that its invasive potential was conferred by the loss of *O*-acetylation in the pneumococcal capsular polysaccharides (20).

Similar to the foreign serotype 35B-CC558 strains, CC558 strains in Japan were found to be associated with multidrug resistance, especially to a broad range of β-lactams, such as penicillin, cephalosporin, and carbapenem. This β-lactam resistance of serotype 35B-CC558 was caused by specific PBP profiles, *pbp1a*-4, *pbp2b*-7, and *pbp2x*-7, shared by global MDR serotype 35B-CC558 isolates (12, 26). Considering the identical CC and PBP profiles, we believe that they share an ancestor and have recently diverged from each other. Our analysis showed that the TMRCA of the strains in Japan was between 1983 and 2001. In particular, our phylogenetic analysis revealed multiple clade formation of the ST558 and ST10493 strains in Japan among the foreign strains, indicating multiple events of introduction of the clone into Japan from foreign countries and vice versa. Our previous studies on global serotype 15A-ST63 (27) and serotype 19A-ST320 (28) and other studies on serotype 3-ST180 (29) and serotype 23F-ST81 (30) demonstrated that pneumococcal clades in phylogenetic trees tended to generate phylogenetic clades based on geographic location. This was especially true in Japan for geopolitical reasons; the country is surrounded by water and foreign migration events are rare. Previous studies suggested that CC558 is associated with multidrug resistance (17, 31) and pili, a pneumococcal virulence factor that facilitates colonization (15, 32, 33). Serotype 35B strains show high potential for biofilm formation *in vitro* (34). These findings suggest that the 35B-CC558 lineage is likely to have a high potential for colonizing the human nasopharynx and being resistant to clearance, which might be associated with a longer duration of carriage and consequent international transmission. Although further studies are needed to support characteristics like the duration of colonization, ability of adhesion, and fitness cost in colonizers, the trend of this clone should be monitored because serotype 35B is not covered by any of the current conjugate vaccines, polysaccharide vaccines, or the upcoming PCV20.

In a previous study, seroconversion to 35D from 35B was suggested to occur independently via the variable point mutations and indels in *wciG* (20). Additionally, in our study, two pairs of the 35D isolates possessed the same mutation/indels in *wciG* and were derived from the same regions, which suggested clonal expansion of the 35D isolates. As discussed above, the 35B-CC558 lineage is suspected to have a high potential for colonizing the human nasopharynx and being resistant to clearance. A recent study showed that within-host microevolution of S. pneumoniae was rapid and adaptive during natural colonization (35), which might facilitate the seroconversion. Considering this result, a longer duration of carriage can also accelerate the nucleotide substitution rate within the host and might lead to seroconversion to 35D. In a previous study, *wciG*-mediated *O*-acetylation seemed to yield an antigenically dominant epitope for serotype 35B, and seroconversion to 35D was associated with the loss of *O*-acetylation, resulting in escape from the acquired immune system response targeting serotype 35B (19). The immune escape might lead to the clonal expansion of the 35D isolates. These findings imply that serotype conversion from 35B to 35D might occur frequently and expand clonally, especially under selective pressure, such as a vaccine covering serotype 35B or immune pressure during colonization. Because limited analysis of 35D isolates was conducted due to the small sample size, further studies are needed to investigate the mechanisms of the serotype conversion.

In addition to the global epidemic CC558 lineage, CC2755 is another prevalent lineage in Japan and was reported predominately in the country (15, 16). CC2755 isolates were associated with multidrug resistance to macrolides, tetracyclines, and trimethoprim-sulfamethoxazole. Macrolide resistance in S. pneumoniae is a major clinical problem because macrolides are commonly prescribed for treating bacterial infections in the upper and lower respiratory tracts, including community-acquired infections (36). In Japan, the proportion of macrolide consumption to total antibiotic consumption in outpatient settings is higher than those in European and North American countries (37). The incidence of macrolide-resistant IPDs was reported to have decreased following PCV introduction in the United States and globally (38, 39). However, in Japan, the rate of macrolide resistance in S. pneumoniae remains very high, >80% in the nationwide surveillance in 2021, even after PCV13 implementation in 2013 (40). In this study, 94.3% of the 35B/D isolates were resistant to erythromycin, and most of the resistance was mediated by *ermB* and/or *mefE* carried on the MEGA in ST10493 (CC558) and on Tn*916*-like ICEs Tn*2009* and Tn*2010* in ST558 (CC558) and Tn*6002* in CC2755. Previously, we found that the predominant ICEs associated with macrolide resistance in 15A-CC63 and 19A-CC3111 strains in Japan were Tn*6002* and Tn*2017*, respectively (41, 42). Recent studies have suggested that Tn*916*-like ICEs, such as Tn*2009*, Tn*2010*, Tn*3872*, Tn*6002*, and the MEGA, are also commonly found in viridans group streptococci colonizing the human throat, mainly Streptococcus mitis, Streptococcus oralis, and Streptococcus parasanguinis (43). Another recent study revealed that high macrolide consumption in the community induced an increase in the prevalence of nasopharyngeal *ermB* and *mefA/-E* resistomes in preschool children (44). These findings imply that the acquisition of macrolide resistance determinants like *ermB* and *mefE* through intra- and interspecies transfer of Tn*916*-like ICEs or MEGAs accelerates the clonal expansion of the strains under the selective pressure of high macrolide consumption. Further studies are needed to elucidate the roles of and relationships between ICEs and the MEGA in macrolide resistance in S. pneumoniae and other commensal bacteria in the nasopharynx.

In conclusion, we conducted whole-genome sequencing to investigate the molecular epidemiology of Japanese serotype 35B/D S. pneumoniae isolates and compared the sequences to previously reported global ones. We revealed multiple events of introduction of the MDR 35B/D-CC558 lineage into Japan, suggesting frequent intercontinental transmission of this lineage. In addition, serotype 35D isolates showed clonality, which suggested potential clonal expansion of the 35D isolates. Our study also revealed that 35B/D isolates in Japan are highly associated with multidrug resistance, including broad-spectrum β-lactam resistance in CC558 and macrolide, tetracycline, and clindamycin resistance via transferable elements, the MEGA, and Tn*916*-like ICEs, in both CCs. Global molecular surveillance utilizing whole-genome-sequencing methods will help to understand the dissemination patterns of the global epidemic clones.

## MATERIALS AND METHODS

### Bacterial isolates.

This study was part of a nationwide prospective surveillance study of pediatric IPDs and non-IPDs in Japan between January 2012 and December 2017 (31, 45). A total of 1,358 pneumococcal isolates were collected from 249 medical institutions across Japan and included 87 serotype 35B and 35D isolates obtained from 30 IPD and 57 non-IPD patients. Among those from the IPD patients, 23 isolates were obtained from blood, 6 from cerebrospinal fluid, and 1 from joint fluid. Non-IPD cases included 50 patients with otitis media and 7 with pneumonia. The detailed characteristics of the isolates are shown in Data Set S1.

### Antimicrobial susceptibility tests.

Susceptibility tests for penicillin G (PCG), erythromycin (EM), cefotaxime (CTX), meropenem (MEM), and levofloxacin (LFX) were performed using the broth microdilution method following the Clinical and Laboratory Standards Institute (CLSI) guidelines (46). The 2015 CLSI standard for categorization (46) was used to determine susceptibility, and isolates identified as having intermediate or full resistance were grouped together as nonsusceptible. We used the CLSI criteria for meningitis for all isolates to allow comparison with previous reports. The following breakpoints were applied for susceptibility, intermediate resistance, resistance, and high resistance, respectively: PCG, ≤0.06, 0.12, 1.0, and ≥2 μg/mL; CTX, ≤0.5, 1.0, 2.0, and >2 μg/mL; MEM, ≤0.25, 0.5, 1.0, and >1 μg/mL; and EM, ≤0.25, 0.5, 16, and >16 μg/mL.

### Whole-genome sequencing and genome analyses.

Whole-genome sequencing was performed on 79/87 of the 35B and 35D isolates in the current study, whereas sequencing data for the other 8 isolates were obtained previously (27). We extracted total genomic DNA using the QIAamp DNA minikit (Qiagen, Hilden, Germany) and prepared sequencing libraries using the Nextera XT DNA library preparation kit (Illumina, San Diego, CA, USA) (27, 41). The details of genome analyses are shown in the supplemental material.

### *In silico* and phenotypic serotyping and *wciG* locus comparison.

We performed *in silico* serotyping using Pathogenwatch (https://pathogen.watch). We performed multilocus sequence typing (MLST) to determine the exact matches for the seven loci (*aroE*, *gdh*, *gki*, *recP*, *spi*, *xpt*, and *ddl*) and assigned clonal complexes (CCs) in agreement with six of the seven loci, with the most predominant sequence type (ST) representing a CC. We extracted sequences of the *wciG* locus from the assembled contigs using BLAST+ version 2.9.0 (47) and the serotype 35B *wciG* reference locus (GenBank accession number KX021817). We performed phenotypic serotyping via the Quellung reaction using pneumococcal typing antisera (Statens Serum Institut, Copenhagen, Denmark) on an overnight culture derived from a single colony. We interpreted the Quellung results as previously described (20).

### PBP typing, antimicrobial resistance gene detection, and GPSC identification.

We extracted the penicillin-binding protein 1A (PBP1A), -2B, and -2X transpeptidase domain sequences of the isolates and assigned PBP transpeptidase domain types using the U.S. Centers for Disease Control and Prevention PBP-type database (https://www.cdc.gov/streplab/pneumococcus/mic.html). PBP types that had not been previously published in the database (last accessed on 1 April 2021) were labeled with the prefix JP (e.g., “*pbp1a*-JP1”). Some of these unassigned PBP types from Japan have been reported (27, 28, 41, 42, 48). Furthermore, we detected *ermB*, *ermTR*, *mefA*, *mefE*, *tetM*, and *tetO* genes and searched for point mutations within the *folA* and *folP* genes in the assembled contigs (41). In addition, we assigned global pneumococcal sequence cluster (GPSC) numbers and detected *tet*(S/M) using Pathogenwatch (https://pathogen.watch).

### Tn*916*-like ICE analysis and *cps* locus comparison.

We extracted the sequences of Tn*916*-like integrative and conjugative elements (ICEs) from the assembled contigs using BLAST+ (47) and an Enterococcus faecalis Tn*916* reference sequence (GenBank accession no. U09422.1). The analyzed sequences were annotated using Prokka version 1.14.6 (49), and the structures of the regions were analyzed manually using the Artemis Comparison Tool (ACT) (50). We also extracted sequences of the *cps* locus from the assembled contigs using BLAST+ and manually analyzed them using ACT.

### Single nucleotide polymorphism (SNP) and phylogenetic analyses.

The core genomes of the 87 serotype 35B and 35D isolates were identified using Prokka version 1.14.6 (49) and Roary version 3.13.0 (51), with standard parameters. A maximum-likelihood phylogenetic tree was generated from the core genome alignment using RAxML-NG version 1.0.3 with a GTR+Γ DNA substitution model (52). To investigate the genetic relationship between the strains previously deposited as the same GPSCs, we obtained sequence data from the European Nucleotide Archive (https://www.ebi.ac.uk/ena/browser/home). A total of 88 strains (GPSC59, 75 strains, and GPSC186, 13 strains) were included for further analysis (Data Set S2).

### Bayesian analysis.

We reconstructed a maximum-credibility clade tree and obtained the dates of ancestors or nodes of the ST558 and ST10493 clades using the Bayesian Markov chain Monte Carlo framework. For this analysis, we performed recombination prediction using Gubbins version 2.4.1 (53). The final SNP alignments, without recombination regions, were used as the input data set for BEAST2 version 2.6.6 (21).

The details of genome analyses are shown in the supplemental material.

### Statistical analysis.

Categorical variables were compared using the χ^2^ and Fisher’s exact tests, as appropriate. A two-sided *P* value of <0.05 was considered to indicate statistical significance. All statistical analyses were performed using R version 3.6.0.

### Ethics statement.

This study was reviewed and approved by the Ethics Committee of Mie Hospital (acceptance number, 23-8). Informed consent for collection and use of patient information and specimens was obtained from each parent/guardian by a primary physician.

### Data availability.

Nucleotide sequence data obtained in this study have been submitted to GenBank/ENA/DDBJ under BioProject accession number PRJDB13244 (DRR356353 to DRR356452) and PRJDB13326 (AP025936 to AP025940, BRKZ01000001 to BRKZ01000009, and BRLA01000001 to BRLA01000013).
