# Supplementary material for: Frequent Transmission of Streptococcus pneumoniae Serotype 35B and 35D, Clonal Complex 558 Lineage, across Continents and the Formation of Multiple Clades in Japan
Source: Antimicrob Agents Chemother. 2023 Jan 18;67(2):e01083-22. doi: 10.1128/aac.01083-22 (PMC9933736; doi:10.1128/aac.01083-22)
Supplement: Supplemental file 4 — Data Set S4. Download aac.01083-22-s0004.pdf, PDF file, 0.97 MB [file aac.01083-22-s0004.pdf]

Frequent transmission of *Streptococcus pneumoniae* serotype 35B/D-CC558 lineage across continents and the formation of multiple clades in Japan

Supplementary Material and Methods

Whole-genome sequencing and genome analyses.

Whole-genome sequencing was performed on 79/87 of the 35B and 35D isolates in the current study, whereas sequencing data for the other eight isolates were obtained previously (1). We extracted total genomic DNA using QIAamp DNA Mini Kit (QIAGEN, Hilden, Germany) and prepared sequencing libraries using Nextera XT DNA Library Preparation Kit (Illumina, San Diego, CA, USA) (1, 2). The samples were multiplexed and sequenced using Illumina NextSeq 500 for 300 cycles (2 ×150-bp paired-end). All data were processed using fastp version 0.20.1 (3) with the following options: -e 25 -n 5 -t 1 -T 1 -l 25 -g -z 4. Trimmed sequences used SPAdes v.3.15.2 (4) for assembly in careful mode with k-mers of 21, 33, 45, 57, 69, 81. 93, 105, 117 and 127. The quality of the assemblies was evaluated using QUAST v5.0.2 (5). In addition, we obtained long sequencing reads from six serotype 35D isolates using the Nanopore MinION platform and the R9.4.1 flow cell (Oxford Nanopore Technologies, Oxford, UK). Base calling was carried out using Guppy v4.3.4 with a high-accuracy model, and hybrid assembly was performed using Unicycler v.0.4.8 (6) with default parameters.

Single nucleotide polymorphism (SNP) and phylogenetic analyses. The core genomes of the 87 serotype 35B and 35D isolates were identified using Prokka v1.14.6 (7) and Roary v3.13.0 (8), with standard parameters. A maximum-likelihood phylogenetic tree was generated from the core genome alignment using RAxML-NG v1.0.3, with a GTR+Γ DNA substitution model (9). To investigate the genetic relationship between the strains previously deposited as the same GPSCs, we obtained sequence data from European Nucleotide Archive (https://www.ebi.ac.uk/ena/browser/home). A total of 88 strains (GPSC59:75 strains, GPSC186:13 strains) were included for further analysis (Supplementary dataset 2). The information of the geographical location of isolation and the host health state (host status) of each strain was obtained through European Nucleotide Archive and EMBL's European Bioinformatics Institute (EBML-EBI, https://www.ebi.ac.uk). Host status was grouped into IPD, non-IPD or carriage. If strains were isolated from sterile sites, such as blood, cerebrospinal fluid and joint fluid, the host status was defined as IPD. The details of genome analyses are shown in the supplementary materials. We used SAMtools v1.12 to identify and remove duplicated regions in the

sequences (10). Thereafter, the fastq data of the GPSC59 isolates were aligned against that for the *S. pneumoniae* serotype 35B strain, Utah 35B-24 (ST377, BAA-660^{TM}[ATCC], accession no. AP025939), whereas those of the GPSC186 strains were aligned against that of *S. pneumoniae* PC1044, using the short-read alignment component of Burrows-Wheeler Aligner (BWA) v.0.7.17 (11). We next identified SNPs and insertions/deletions using Genome Analysis Toolkit (GATK) v4.1.9, following Best Practices Workflows (12). Recombination sites were assessed using Gubbins v2.4.1 (13) and a recombination site-censored maximum-likelihood tree was generated using RAxML-NG v1.0.3 (9). The nodes were supported by 100 bootstrap replicates. The phylogenetic trees were visualized using FigTree v1.4.4 (available at https://github.com/rambaut/figtree/) and iTOL v6 (14). We defined the clade including ≥ 5 Japanese isolates on the phylogenetic tree as a major cluster and the clade including < 5 isolates as a minor cluster.

Bayesian analysis. We reconstructed a maximum clade credibility tree and obtained the dates of ancestors or nodes of the ST558 and ST10493 clades using the Bayesian Markov Chain Monte Carlo framework. For this analysis, we performed recombination prediction using Gubbins v2.4.1 (13). The final SNP alignments, without recombination regions, were used as the input dataset for BEAST2 v2.6.6 (15). For examination of the temporal signal of the inputted dataset, we used TempEst v1.5.3 (16) to diagnose the regression of the root-to-tip genetic distance against the sampling time. Analysis of the best-fitting root mean square of the heuristic residual showed that the correlation coefficients (R^2) of all isolates were 0.4066. We used strict exponentially relaxed and lognormal-relaxed molecular clocks with constant-size coalescent and Bayesian skyline coalescent, respectively. Convergence and mixing were checked using Tracer v1.7.2. For each model, to obtain an effective sample size (ESS) greater than 200 for all factors, we set the MCMC lengths to 100 million. We specified a general time-reversible substitution model with site rate heterogeneity modeled across four gamma distributions (GTR+Γ4) for all analyses. The model was selected through comparisons of the marginal likelihood using path sampling and stepping stone-based marginal likelihood estimation. For each setting, we used default priors in BEAUti v2.6.6. According to the results, we selected a strict clock model and constant size prior model for the analysis. The phylogenetic tree was visualized using FigTree v1.4.4 (available at https://github.com/rambaut/figtree/).

Supplementary Table 1. Antimicrobial susceptibility and PBP profiles of serotype 35B/D isolates in Japan.

CC[a] (no.)	PBP[b] profile 1a:2b:2x (no.)	MIC[c] (mg/liter)																
		PCG[d]						CTX[d]						MEM[d]				
		<0.06	0.12	0.25	0.5	1.0	2.0	≤0.12	0.25	0.5	1.0	2.0	4.0	<0.06	0.12	0.25	0.5	1.0
558 (67)	4:7:7 (66)	0	0	0	1	43	22	0	1	32	33	0	0	0	0	1	61	4
	4:7:JP46 (1)	0	0	0	0	0	1	0	0	0	0	0	1	0	0	0	1	0
2755 (20)	0:0JP34 (5)	5	0	0	0	0	0	0	5	0	0	0	0	5	0	0	0	0
	0:0:36 (3)	3	0	0	0	0	0	0	1	2	0	0	0	3	0	0	0	0
	0:0:JP37 (3)	3	0	0	0	0	0	0	3	0	0	0	0	3	0	0	0	0
	0:0:73 (2)	2	0	0	0	0	0	0	1	1	0	0	0	2	0	0	0	0
	others (7)	6	1	0	0	0	0	2	2	2	1	0	0	7	0	0	0	0
Total		19	1	0	1	43	23	2	13	37	34	0	1	20	0	1	62	4

[a] CC: Clonal complex

[b] PBP: Protein-binding protein

[c] MIC: Minimum inhibitory concentration

[d] PCG: Penicillin G, CTX: Cefotaxim, MEM: Meropenem

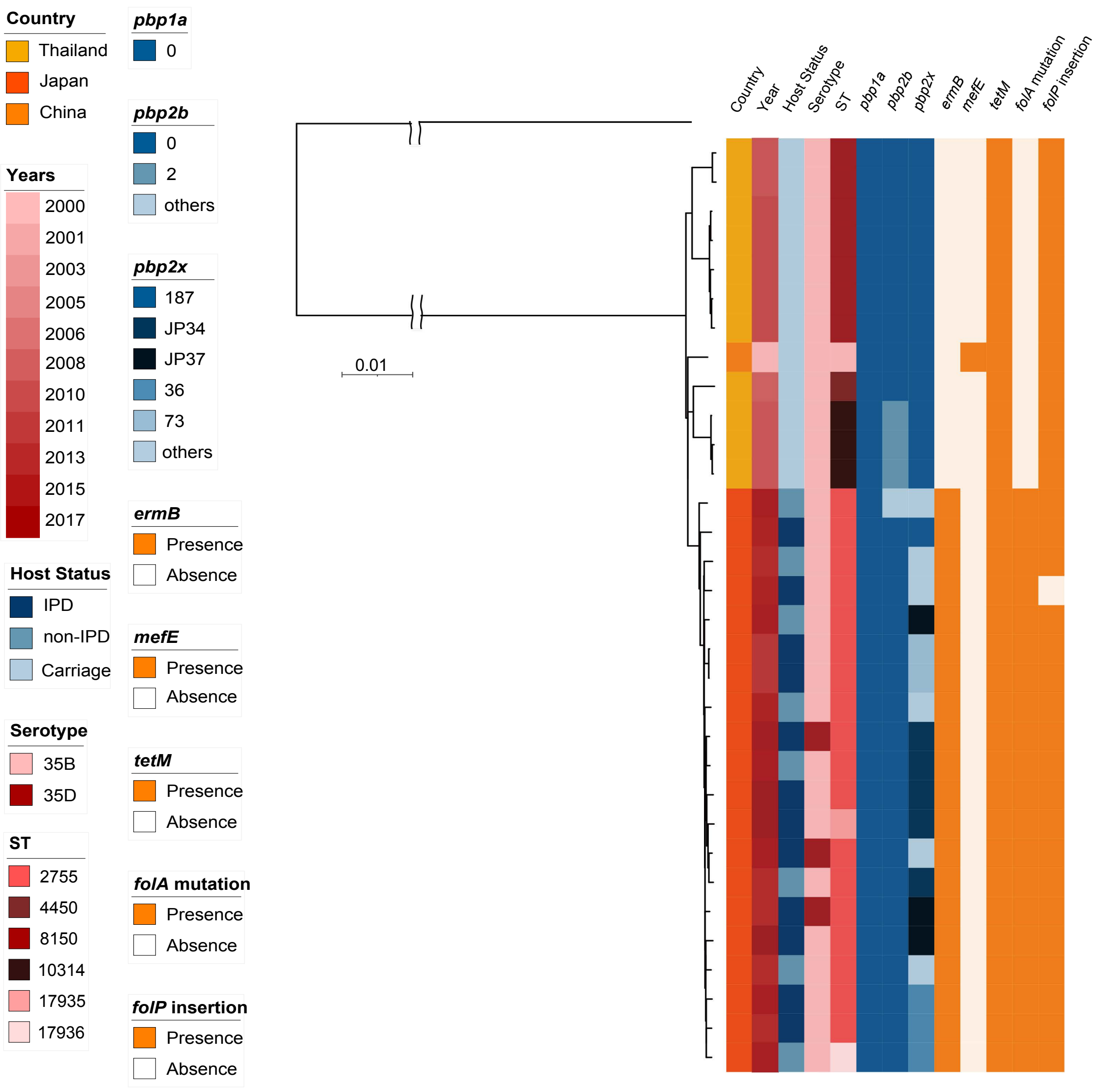

Supplementary Figure 1. Recombination-free maximum-likelihood tree of the GPSC186 isolates in Japan and previously deposited isolates in the NCBI database, rooted in the distantly related *S. pneumoniae* isolate ERS1299868. Color bars indicate the country from which the isolate was obtained; year of isolation; host status; serotype; sequence type; type of *pbp1a*, *php2b*, *pbp2x*; and presence of antimicrobial resistance determinants, *ermB*, *tetM* and *folA* I100L mutation and *folP* insertion.

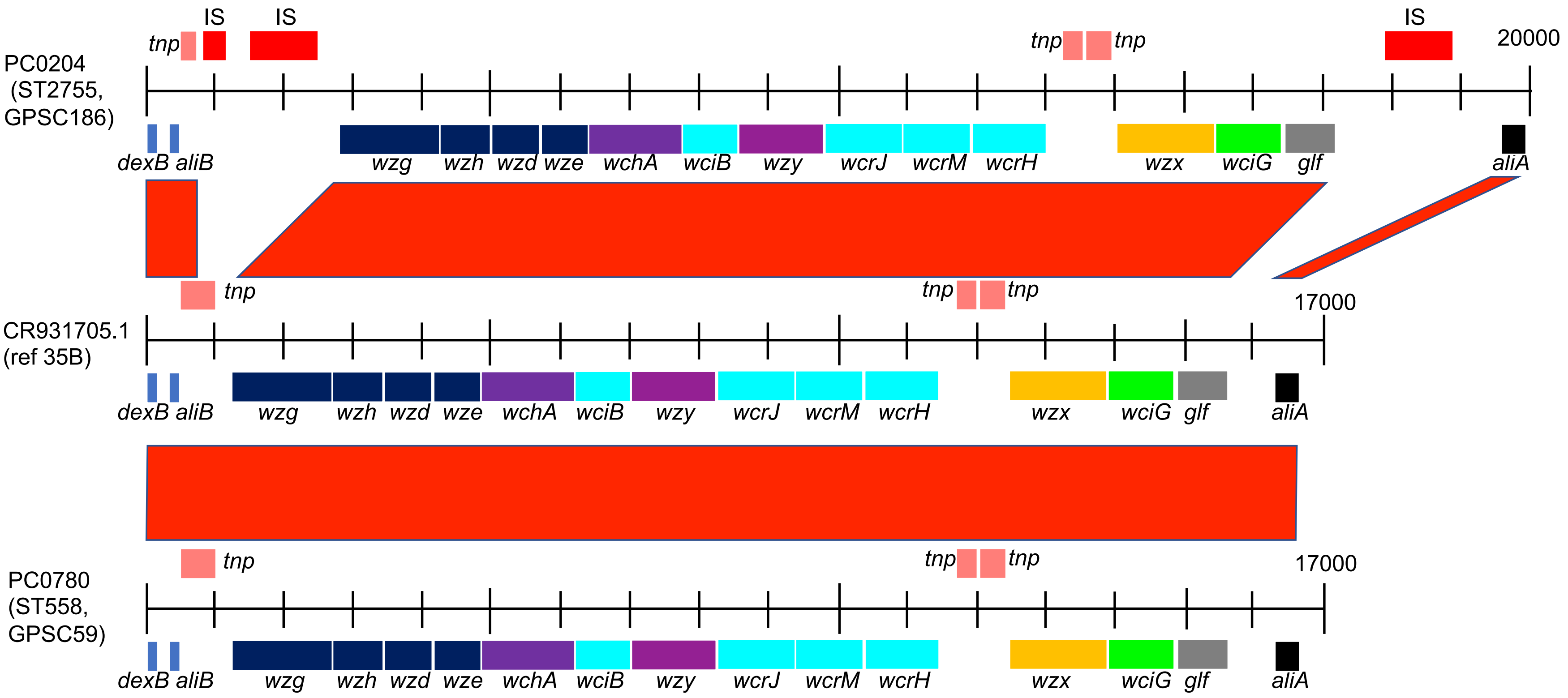

Supplementary Figure 2. Comparison of cps regions of ST558 and ST2755 isolates in Japan. Red bands between the sequences indicate BLASTN matches. The reference sequence of *cps* was submitted to the NCBI reference sequence CR931705.1

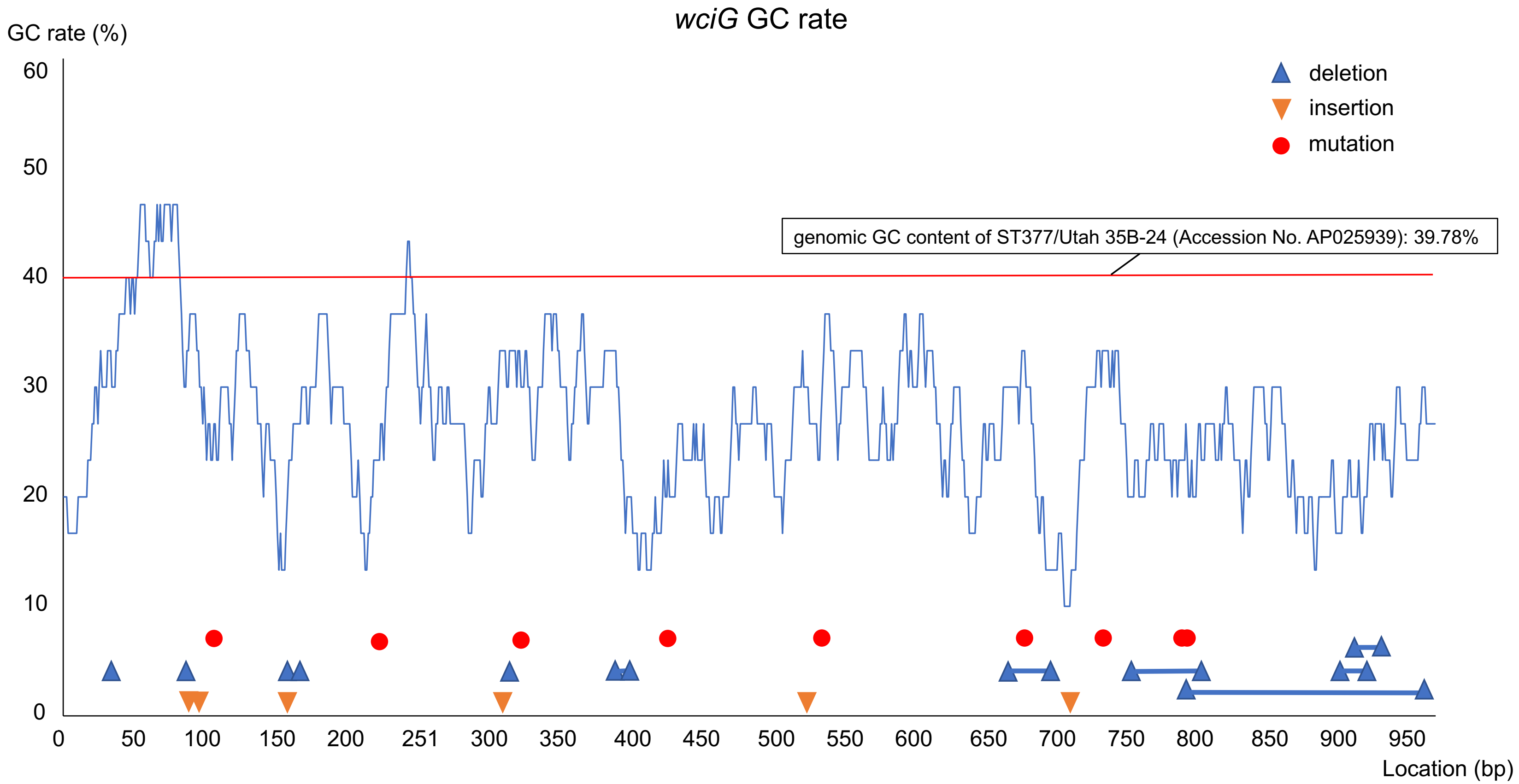

Supplementary Figure 3. Location of deletion, insertion, and mutations associated with seroconversion to 35D in our isolates and previously reported isolates, along with the GC rates with a 30 bp width in each position. Red line represents the genomic GC content of Utah 35B-24 (ST377, BAA-660TM[ATCC]; accession no. AP025939).

Supplementary References

1. Nakano S, Fujisawa T, Ito Y, Chang B, Matsumura Y, Yamamoto M, Nagao M, Suga S, Ohnishi M, Ichiyama S. 2018. Spread of Meropenem-Resistant *Streptococcus pneumoniae* Serotype 15A-ST63 Clone in Japan, 2012-2014. Emerg Infect Dis 24:275–283.
2. Nakano S, Fujisawa T, Ito Y, Chang B, Matsumura Y, Yamamoto M, Suga S, Ohnishi M, Nagao M. 2019. Penicillin-Binding Protein Typing, Antibiotic Resistance Gene Identification, and Molecular Phylogenetic Analysis of Meropenem-Resistant *Streptococcus pneumoniae* Serotype 19A-CC3111 Strains in Japan. Antimicrob Agents Chemother 63:e00711-19.
3. Chen S, Zhou Y, Chen Y, Gu J. 2018. fastp: an ultra-fast all-in-one FASTQ preprocessor. Bioinforma Oxf Engl 34:i884–i890.
4. Prjibelski A, Antipov D, Meleshko D, Lapidus A, Korobeynikov A. 2020. Using SPAdes De Novo Assembler. Curr Protoc Bioinforma 70:e102.
5. Gurevich A, Saveliev V, Vyahhi N, Tesler G. 2013. QUAST: quality assessment tool for genome assemblies. Bioinforma Oxf Engl 29:1072–1075.
6. Wick RR, Judd LM, Gorrie CL, Holt KE. 2017. Unicycler: Resolving bacterial genome assemblies from short and long sequencing reads. PLOS Comput Biol 13:e1005595.
7. Seemann T. 2014. Prokka: rapid prokaryotic genome annotation. Bioinformatics 30:2068–2069.
8. Page AJ, Cummins CA, Hunt M, Wong VK, Reuter S, Holden MTG, Fookes M, Falush D, Keane JA, Parkhill J. 2015. Roary: rapid large-scale prokaryote pan genome analysis. Bioinforma Oxf Engl 31:3691–3693.
9. Kozlov AM, Darriba D, Flouri T, Morel B, Stamatakis A. 2019. RAxML-NG: a fast, scalable and user-friendly tool for maximum likelihood phylogenetic inference. Bioinforma Oxf Engl 35:4453–4455.
10. Danecek P, Bonfield JK, Liddle J, Marshall J, Ohan V, Pollard MO, Whitwham A, Keane T, McCarthy SA, Davies RM, Li H. 2021. Twelve years of SAMtools and BCFtools. GigaScience 10:giab008.
11. Li H, Durbin R. 2009. Fast and accurate short read alignment with Burrows-Wheeler transform. Bioinforma Oxf Engl 25:1754–1760.
12. McKenna A, Hanna M, Banks E, Sivachenko A, Cibulskis K, Kernytsky A, Garimella K, Altshuler D, Gabriel S, Daly M, DePristo MA. 2010. The Genome Analysis Toolkit: a MapReduce framework for analyzing next-generation DNA sequencing data. Genome Res 20:1297–1303.

13. Croucher NJ, Page AJ, Connor TR, Delaney AJ, Keane JA, Bentley SD, Parkhill J, Harris SR. 2015. Rapid phylogenetic analysis of large samples of recombinant bacterial whole genome sequences using Gubbins. Nucleic Acids Res 43:e15.
14. Letunic I, Bork P. 2021. Interactive Tree Of Life (iTOL) v5: an online tool for phylogenetic tree display and annotation. Nucleic Acids Res 49:W293–W296.
15. Bouckaert R, Vaughan TG, Barido-Sottani J, Duchêne S, Fourment M, Gavryushkina A, Heled J, Jones G, Kühnert D, De Maio N, Matschiner M, Mendes FK, Müller NF, Ogilvie HA, du Plessis L, Popinga A, Rambaut A, Rasmussen D, Siveroni I, Suchard MA, Wu C-H, Xie D, Zhang C, Stadler T, Drummond AJ. 2019. BEAST 2.5: An advanced software platform for Bayesian evolutionary analysis. PLoS Comput Biol 15:e1006650.
16. Rambaut A, Lam TT, Max Carvalho L, Pybus OG. 2016. Exploring the temporal structure of heterochronous sequences using TempEst (formerly Path-O-Gen). Virus Evol 2:vew007.
